# Can cumulative disadvantages be reversed? Class attainment and a network analysis of intergenerational occupational pathways by migratory origin in Buenos Aires, Argentina

**DOI:** 10.3389/fsoc.2026.1755911

**Published:** 2026-05-21

**Authors:** Pablo Dalle, Joaquín Carrascosa

**Affiliations:** 1 CONICET, Buenos Aires, Argentina; 2 Instituto de Investigaciones Gino Germani, Universidad de Buenos Aires, Buenos Aires, Argentina

**Keywords:** ethnicity, gender, intergenerational social mobility, migration, social class, social network analysis (SNA)

## Abstract

This article analyzes patterns of intergenerational social mobility by family migratory origin in the Buenos Aires Metropolitan Area. The Argentine case contributes to current debates on assimilation and social mobility by comparing long-term class attainment among descendants of European immigrants and those of internal (“mestizo”) or Latin American origin. Drawing on pooled data from two recent probabilistic surveys (2021 and 2023), we apply multinomial logistic regression models to estimate the relative chances of social class attainment across different groups. In addition, social network analysis (SNA) is employed to conduct a micro-level examination of intergenerational occupational trajectories among migrants, natives, and their descendants, who share similar working-class backgrounds but differ in ethnic origin. This approach highlights occupations that serve as springboards for upward mobility and magnets for class reproduction. Results show that migrants, both internal and regional, have had fewer opportunities for upward mobility. Second-generation individuals born in the Buenos Aires Metropolitan Area (MABA) of mestizo descent and children of Latin American migrants display similar chances of upward mobility to those of European descent when controlling for class origins and education. However, the children of internal migrants of mestizo descent exhibit limited upward mobility, even after controlling for class origins and educational attainment. This pattern is also observed among the first generation of internal migrants and among Latin American migrants. Disparities in class attainment reflect the cumulative disadvantages experienced by subaltern ethnic groups, rooted in long-standing structural inequalities. However, the micro-level analysis shows that, among disadvantaged migrant groups, the offspring of Latin American immigrants tend to experience slightly greater short-range upward mobility, particularly through gendered patterns, such as manual crafts among men and tertiary qualifications among women, especially in health and education.

## Introduction

The interrelationship between migration flows, ethnic origin, and opportunities for upward social mobility constitutes a central issue in national debates on the constitutive features of contemporary Argentine society. For a long time, Argentina imagined itself as a “melting pot.” This metaphor emphasized that modern Argentine society emerged from the mixture of European immigrants, criollos (mestizos), and Indigenous populations, while rendering Afro-descendant populations largely invisible, giving rise to a new culture and an integrated nation built upon socio-cultural diversity. Within this imaginary, Argentina was portrayed as a non-racist society, lacking the overt racial conflicts associated with countries such as the United States or South Africa. At the same time, the “melting pot” narrative suggested an open society in which access to upward mobility was not strongly constrained by ascriptive characteristics such as ethnic origin.

However, this fiction of “integration” conceals profound ethnic diversity and hierarchies linked to racialized phenotypical traits that serve as sources of inequality. The melting pot ideal has been critically reassessed by numerous studies. Unlike in other Latin American countries such as Mexico or Peru, Argentina never developed a hegemonic national model based on *mestizaje* that transformed hybridity and the figure of the mestizo into symbolic capital of national identity. Instead, the melting pot myth implied the subordinate assimilation of mestizo and Indigenous populations. This imaginary projected an image of a white and European Argentina, in which incorporation was conditional upon the whitening of subaltern ethnic groups, including large sectors of the mestizo population and Latin American migrants (Briones, 2002; Margulis and Urresti, 1999; Garguin, 2007; Adamovsky, 2012; Grimson and Karasik, 2021; Caggiano, 2023).

While these studies have extensively documented the “melting pot” narrative and its critiques, they have paid far less attention to the extent to which ethnic descent constitutes a source of inequality in opportunities for upward social mobility across generations. This study addresses this gap by examining the effects of migratory origin, considering both migrant generation and ethnic descent, in order to capture the interplay of socio-cultural factors and patterns of economic development that shape intergenerational mobility processes. Empirically, the analysis focuses on the Buenos Aires Metropolitan Area, the country’s main economic center and the primary destination of historical and contemporary migratory flows, which makes it a distinctly multiethnic metropolis.

The goal of this paper is to analyze intergenerational social mobility patterns in the population of the Buenos Aires Metropolitan Area (MABA) by family migratory origin, in relation to the main migration streams to this metropolis: overseas (mostly European), internal migrants of *criollo*^1^*/mestizo* descent, and migrants from other Latin American countries.

Both migration research and studies of intergenerational social mobility analyze the influence of ascriptive factors, that is, inherited circumstances, on life chances; however, these two lines of research have largely developed along parallel tracks. On the one hand, several studies on migrants, based on occupational rate calculations, have documented the disadvantages faced by Latin American migrants in the labor market, including higher levels of informality and lower wages, as well as the socio-economic profiles of migrant populations (Maguid, 2011; OIT and OIM, 2025; Cerrutti, 2018, among others). However, these studies do not address the effect of social origin (i.e., parental social class or educational level) on these outcomes. Other predominantly qualitative studies focus on the productive activities, forms of sociability, and cultural practices that shape the ethnic enclaves of the main migratory groups in the Buenos Aires Metropolitan Area (MABA): the Bolivian enclave, linked to horticultural activities (Benencia, 2012), as well as small textile workshops and itinerant trade (Parra, 2021); the Paraguayan enclave, associated with the construction sector (Del Águila, 2016); and the Peruvian enclave, with a strong presence in popular retail commerce (Herrera Jurado, 2022).

On the other hand, studies of intergenerational social mobility in Argentina have identified a general pattern of persistent fluidity (Jorrat and Benza, 2016), alongside an increasing closure of the class structure regarding upward mobility from working-class origins (Dalle, 2018). However, the influence of both international and internal migration, as well as ethnic origin, on patterns of intergenerational mobility—a line of inquiry initiated by Germani (1963)—has received limited attention in recent decades, aside from our own research program (Dalle, 2016, 2025; Dalle and Herrera Jurado, 2024).

Regarding previous research in the field of social stratification in Argentina, this paper aims to contribute in three areas: (i) the inclusion of migratory origins and ethnic descent as central ascriptive factors that shape inequality of opportunities, (ii) the combined use of these variables to quantify the effects of hardships or advantages linked to family migratory origin on different paths of upward social mobility, controlling for class origin and educational attainment; (iii) the identification of specific occupational channels of upward mobility and class reproduction across different migratory groups by gender at a micro level.

The main guiding questions of our research are: i. Did the first generation of migrants from different backgrounds face penalties in terms of upward class mobility in the second decade of the 21st century? If such inequalities in opportunities are found, have they persisted among their descendants? To what extent are the effects of migratory origin and ethnic descent explained by their association with social class origin, based on the historical accumulation of disadvantages? Do persistent effects remain in the present when controlling for social class origin and educational attainment? Is it possible to identify gender differences in these effects? If there are additional penalties for subaltern migratory groups, what are their structural and cultural roots? What are the main channels of upward mobility and class reproduction from working-class origins across migratory origins, and how are they structured by gender?

First, we analyze the evolving role of class origins and education in the social mobility processes of groups with different migratory backgrounds. By using multinomial logistic regressions (Treiman, 2009), we examine variations in the effects of class origins and education on a categorical outcome variable representing different mobility paths. Then, we deepen the analysis of micro-level examination of intergenerational occupational pathways through social network analysis (SNA) (Cheng and Park, 2020; York et al., 2025), highlighting both upward and downward mobility, as well as class reproduction from working-class origins by gender. Specifically, we analyze occupations that function as *magnets* for class reproduction and those that serve as *springsboards* for upward mobility among both migrants and natives, as well as their descendants who share similar working-class backgrounds.

### Theoretical background

Do migrants and their adult children succeed in overcoming the disadvantages of their origin**s**?

First-generation migrants, while often positively selected in terms of education and motivation for upward mobility in their contexts of origin, frequently encounter labor market disadvantages and enter at the bottom of the class structure. While this pattern is common among different migratory origins, it is also true that they transmit a strong disposition for social upward mobility to their sons and daughters. Currently, there is an interesting debate about the opportunities for upward mobility among migrants’ adult children, which can be framed within two main perspectives (Ferry and Ichou, 2024): On the one hand, classical linear assimilation (Duncan and Duncan, 1968) or Neo-assimilation (Alba and Nee, 2003; Alba et al., 2011) suggest that while migration matters initially, no “migration penalty” persists for subsequent generations, leading to convergence with mainstream society. On the other hand, “segmented assimilation” (Portes and Zhou, 1993) and “pluralist incorporation” (Luthra and Waldinger, 2013) propose that the effects of migration origin can have either a negative or positive impact across immigrant communities (ethnicities).

In the United States, the structural-functionalist school developed the theory of linear assimilation, which posits that sociocultural assimilation fosters upward social mobility. Classic studies showed that among European immigrant minorities, once social origin and educational attainment were held constant, no substantive inequalities in status attainment remained (Duncan and Duncan, 1968). However, this openness of the social structure applied only to the white population, as Afro-descendants remained largely confined to the reproduction of working-class positions, even when achieving higher levels of education, highlighting the persistence of racial discrimination (Blau and Duncan, 1967).

Contemporary studies focused on the offspring of subaltern ethnic groups have challenged the linear assimilation thesis in the context of increased ethnic discrimination and limited job opportunities in middle classes. The theory of segmented assimilation proposes a typology of trajectories for ethnic minorities: (i) classical assimilation leads to long-range upward social mobility and cultural integration into the upper-middle classes; (ii) dissonant assimilation, characterized by a rupture with one’s original culture, exposes individuals to downward mobility, discrimination, and marginalization; and conversely (iii) the valorization of ethnic capital and community ties can foster resilience against inequalities and social exclusion, promoting short-range upward mobility trajectories (Portes and Zhou, 1993; Portes and Rumbaut, 2006).

This study focuses exclusively on the structural dimension of assimilation. Within the framework of linear assimilation, this dimension entails an intergenerational process of upward social mobility among migrants and their descendants, meaning movement into the middle and upper-middle classes and convergence with, or even improvement upon, the social patterns of the native majority. In contrast, the segmented assimilation or pluralist incorporation perspectives emphasize that migrant groups with subaltern ethno-racial origins often experience fewer opportunities for upward mobility and follow distinct pathways of socioeconomic advancement, typically linked to enclave economies that remain largely separate from the mainstream middle and upper-middle classes.

In this vein, a number of studies show that the offspring of subaltern migrant groups experience lower rates of upward social mobility compared to natives and to the children of other migrant-origin groups. This is the case, for instance, for Latin American-origin populations, especially Mexicans, Central Americans, and non-White Caribbeans, in the United States (Portes and Rumbaut, 2006), as well as for the adult children and even grandchildren of North African migrants in several European contexts such as France (Ferry et al., 2025), and for Turkish-origin populations in Germany, as well as Latino and African American populations in the United States (Hertel and Groh Samberg, 2014).

The studies cited above suggest that their disadvantages in class attainment stem from the interaction of multiple structural factors, including processes of racialization within the host society, settlement in socioeconomically disadvantaged and more segregated neighborhoods, and attendance at lower-quality schools (Ferry and Ichou, 2024).

In Latin America, research on stratification and intergenerational mobility has more frequently incorporated ethnic and racial markers of ascription, but has seldom considered migratory background or migrant generation, particularly concerning internal migration. Most studies have documented a segmented and pigmentocratic social structure (Telles and PERLA, 2014), in which individuals who self-identify as having Indigenous descent, darker skin, as Black, or with mixed ethnic backgrounds face disadvantages in access to educational, occupational, and economic positions, inequalities that tend to be more pronounced among women than men (Costa Ribeiro, 2007; Dalle, 2018; Telles et al., 2015; Solís and Boado, 2016, among others).

Regarding the gender dimension, the sex composition of migration to Argentina has changed markedly over time. While the early stages of overseas migration in the late nineteenth century were characterized by a strong male predominance, contemporary migration is marked by a clear feminization of the foreign-born population. The share of women among the foreign-born population increased from 28.5% in 1869 to 54.9% in 2022, with even higher levels in the MABA (National Population and Housing Census, 2022).

The strong presence of women in certain migratory flows, particularly from Paraguay and Peru, is closely linked to the demand for labor in domestic service and care work, serving upper- and upper-middle-class households, as well as broad middle sectors facing difficulties in reconciling work and family life in a context of insufficient public care provision (Cerrutti, 2018). Among Venezuelan migrants, the proportion of women is also higher, although less markedly than in these other groups.

Migrant women have shifted from family reunification roles to central economic agents, driven by a “care crisis” and transnational reproductive labor (Parreñas, 2001). However, low-wage, informal care work (Anderson, 2000) combined with transnational obligations and remittances (Parreñas, 2001; Oso, 2022) creates structural constraints. This limits resource accumulation and perpetuates intergenerational disadvantage.

At the same time, research also points to the existence of upward mobility trajectories among a segment of migrant women from working-class backgrounds who are employed in domestic service. A proportion attain higher levels of tertiary or university education and enter more professionalized segments of care work, particularly nursing, which in some cases may serve as a channel for relative upward mobility within the care sector itself (Mallimaci Barral and Magliano, 2024). Finally, comparative research on the children of migrants in international contexts shows that daughters of immigrants often achieve higher educational outcomes than their brothers and, in several contexts, demonstrate stronger intergenerational educational mobility relative to their parents (e.g., OECD, 2018). These patterns raise the question of whether similar gendered patterns of occupational mobility among migrant women and their daughters are observed in Argentina.

In sum, migrants’ class attainment depends on skills, local development, and gender-structured occupational niches, as well as on the permeability of the “color line” with respect to ethnic/phenotypic distance (Alba, 2005).

### Stages of economic development and migration streams to the MABA: effects on class structure composition

During the period from 1880 to 1930, characterized by an agro-export model of development and the beginnings of industrialization, Argentina’s social structure, particularly in the Pampas region and its epicenter, Buenos Aires, underwent a profound transformation. The country shifted from a polarized and relatively closed society to a more modern and open one, in which the expansion of the middle and urban working classes fostered broad processes of upward mobility from popular origins. European migrants, most of whom were from the working class, and their descendants played a key role in these upward mobility flows (Germani, 1963; Sautu, 1969).

During the 1930–1970 period, the industrial development to substitute import (ISI) goods prompted an intense internal migratory movement toward urban centers, mostly to the MABA (de Recchini Lattes and Lattes, 1969). From a sociocultural perspective, these internal migration flows, especially those of the 1960s originating from provinces with lower levels of prior economic development and less European immigration, brought together two different ethnic groups: the population of mestizo descent and the population of European origin. Unlike the 1860–1930 period, European migration that had contributed to the formation of the middle classes, along with internal migrants and the latest wave of European migrants, joined the working class. The vast majority had been, in their places of origin, semi- or non-qualified manual workers, thus experiencing intergenerational upward social mobility through their integration into industry as salaried laborers or craftsmen (Germani, 1963).

The arrival in the MABA of internal migrants from Argentina’s most economically stagnant regions, as well as from neighboring countries (Paraguay, Bolivia, and Chile), coincided with the second stage of import substitution industrialization (ISI) (1960–1976). During this period, the manufacturing industry underwent a process of concentration that limited opportunities for social mobility among these immigrants, whether through small-scale capital ownership or access to skilled labor positions. The main exceptions were the construction and service sectors, both semi-skilled and low-skilled, whose continued expansion provided important channels for labor market incorporation (Lattes and Sautú, 1978).

The transformation of Argentina’s development model from an industrialization-oriented economy to a more liberalized one, characterized over the past 50 years by strong processes of corporatization and financialization, has contributed to a polarization of the occupational structure. It has involved a major expansion of service-sector occupations, both skilled (managers, professionals, and technicians) and unskilled (routine white-collar employees and manual workers in personal services). On one hand, this trend indicates an increase in opportunities for the upper-middle and middle classes; on the other, it has entailed a shift of labor from skilled manufacturing positions toward lower-skilled jobs in commerce and personal services, characterized by high levels of informality, particularly among women (Sautu, 2016; see also the effects on origin and destination class distributions in Dalle, 2018, p. 27). Upon arrival at the MABA, the main recent external migratory flows (Paraguayan, Bolivian, Venezuelan, Peruvian) have primarily taken up low-wage manual occupations with precarious working conditions in construction, the textile industry, small shops or street commerce, and personal services (mainly domestic work and house cleaning), as well as gastronomy (Cerrutti, 2018). This situation is similar among internal migrants of working-class origin (Dalle, 2025).

## Data and methodology

We pool data from two recent datasets (2021 and 2023);^2^ the PISAC-COVID 2021 survey on “Social structure and public policy from Argentina” and the IIGG-UBA/UNGS 2023 survey on “Occupational trajectories and class formation”. The study population consists of individuals aged 25 to 64 residing in private households in the MABA in 2021^3^ and 2023. The sample is probabilistic, includes 1,638 cases (excluding missing values), and is representative of the MABA population.

The surveys include educational and occupational indicators for two generations (Respondents and heads of household when they were 15 years old, mostly fathers). For both surveys (PISAC-COVID and IIGG-UBA/UNGS), we relied on disaggregated birth-location data across two generations, combined with self-identified ethnic origin, to operationalize ethnic descent.^4^

The class scheme used to measure intergenerational social mobility is based on an aggregation of the Erikson-Goldthorpe-Portocarero (EGP) class schema, adapted to the Latin American context. This adaptation involves reclassifying a segment of the self-employed population from the petite bourgeoisie into the unskilled working class to better capture structural heterogeneity in self-employment under conditions of high labor informality (Solís and Boado, 2016). The resulting class categories are: (1) I–II: Service class; (2) IIIa–IIIb: Routine non-manual employees; (3) IVa–IVb: Petite bourgeoisie (self-employed with and without employees); (4) V–VI: Skilled working class; (5) VIIa–VIIb and IVc: Unskilled working class and agricultural self-employed workers.

The analytical strategy begins with the elaboration of bivariate tables that show the position in the class structure by family migratory origin. We then perform a multinomial logistic regression following a multivariate stepwise analysis to identify the effect of each variable while controlling for the other independent variables (Treiman, 2009). The multinomial logistic model allows us to examine different channels of intergenerational mobility by comparing the relative chances of attaining each class destination versus remaining in, or falling into, the unskilled working class. We use the unskilled working class as the reference category because it represents the bottom of the class structure and provides a meaningful benchmark for assessing different types of mobility, specifically different types of upward mobility from working-class origins.

We adjust a nested regression model, performing hypothesis testing procedures for each group of variables included at each step. The Lr2 test allows us to determine whether the introduction of a new variable or a group of variables is theoretically relevant to the study of social mobility processes and could add statistically significant effects in relation to a simpler model, thus indicating the model’s parsimony (Long and Freese, 2006).

Table 1 shows the univariate distribution for all variables included in the regression models. Migration background and ethnic descent are reconstructed by combining the migratory origins and the ethnicity of the respondents (see Table A1, Supplementary Table 1). This typology allows us to capture the main migratory flows to MABA. In particular, it distinguishes between internal migrants and the offspring of internal migrants by ethnic descent, reflecting distinct historical patterns of internal migration to MABA. Earlier waves of internal migrants were predominantly of European descent and largely originated from provinces in the Pampa Húmeda, which have historically been characterized by higher levels of economic development. By contrast, migrants of Mestizo descent arrived later and tend to originate from more peripheral provinces, characterized by lower levels of overseas migration and more limited economic development (de Recchini Lattes and Lattes, 1969). This latter group is also more exposed to ethno-racial discrimination.

**Table 1:** Description of variables used (%).

Variable	%
Class destinations	
I + II Service class	26.3
IIIab. Routine non-manual class	21.1
Ivab. Petty bourgeoisie	13.2
V + VI. Skilled working class	12.1
VIIab+IVc. Unskilled working class and farm workers	27.3
Class origins	
I + II Service class	18.8
IIIab. Routine non-manual class	13.7
Ivab. Petty bourgeoisie	14.7
V + VI. Skilled working class	16.6
VIIab+IVc. Unskilled working class and farm workers	36.2
Sex	
Men	46.7
Women	53.3
Migration background and ethnic descent	
Second generation born in MABA, European descent	29.2
Born in MABA, European migrant parents	6.2
Born in MABA, internal migrant parents, European descent	17.6
Internal migrants, European descent	3.0
Second generation born in MABA, Mestizo descent	15.5
Born in MABA, Internal migrant parents, Mestizo descent	13.5
Internal migrants, Mestizo descent	4.1
Born in MABA, Latin American migrant parents	5.7
Latin American migrants	5.1
Education	
Less than high school (ref.)	36.3
High school	43.2
Higher education	20.6
Total	1926

Source: pooled surveys (21–23).

### Limitations

This study has some limitations that should be acknowledged. First, the analysis relies on cross-sectional survey data, which limits the ability to directly observe intergenerational processes as they unfold over time and constrains causal inference. As a result, the findings should be interpreted as associations between migratory origin, ethnic background, and educational attainment and class outcomes, rather than as causal effects.

However, the use of retrospective information on parental characteristics (class origin, migratory origin, and ethnic descent) provides a consistent approximation of intergenerational class mobility patterns, a widely used strategy in the field. Within this framework, the analysis aims to identify inequalities across groups, offering valuable insights into how migratory backgrounds and ethnic descent are associated with stratification outcomes in contemporary Buenos Aires.

### Using social network analysis (SNA) to study intergenerational channels of upward mobility and class reproduction

Intergenerational social mobility has traditionally been analyzed with the *mobility table* using methods such as the analysis of inflow and outflow percentages for social classes (usually with 7th class positions or 3 large social classes), log-linear models, and regression models. We complement the multinomial logistic regression approach with a social network analysis (SNA) method to examine intergenerational occupational mobility at the micro level (occupations with ISCO-08 codes at the 4-digit level) (ILO, 2008). This allows us to find subtler patterns of mobility for each family’s migratory origin that might not be as easy to observe with the “macro class” approach. We focus specifically on respondents from working-class origins, analyzing differences by family migratory origin and gender.

Recently, the micro-class approach to the study of social mobility has gained traction by highlighting the key role played by occupations in transferring intergenerational advantages and disadvantages. Macro-class (or big-class) approaches usually focus on the intergenerational reproduction of resources such as *human capital* and *cultural capital* (through general or abstract skills and cultural tastes), *social capital* (through class-wide networks from neighborhood ties, schools, job-related interactions), and *economic resources* (wealth, income, etc.). The micro-class approach highlights occupation-specific types of reproduction for these resources (Jonsson et al., 2009, p. 7). The reproduction of *human capital* involves occupation-specific skills (carpentry, plumbing, acting, etc.), *cultural capital* through occupation-specific cultural tastes (aspirations to become a doctor), *social capital* gained in occupation-specific networks through family or on-the-job interactions, and *fixed economic resources* (businesses, properties, farms, etc.).

Occupational networks have been used to study labor markets and social mobility in the patterns formed by occupations that require similar skills, revealing a trend of polarization between manual and non-manual occupations (Alabdulkareem et al., 2018), as well as examining occupational trajectories and intragenerational mobility (Cheng and Park, 2020; Sautu et al., 2022). We adapt this approach to analyze intergenerational occupational flows from working-class origins, aiming to identify nodes of class reproduction that function as magnets and those that act as springboards for upward social mobility.

To transform survey data into network data, we use the edgelist format. This format treats each occupation from the ISCO-08 at the 4-digit level as a *node*, and these nodes are connected by a *tie* when a respondent reports the occupation of their head of household at age 15 (usually their father and occasionally their mother) alongside their own occupation. We use directed ties (represented as arrows pointing from parents to respondents’ occupations) weighted according to how many times that transition occurs in our survey (i.e., the weight is three if three respondents work as office clerks whose parents worked as bricklayers in our survey).

## Results

### Patterns of class incorporation by migratory origin and ethnic descent

What kinds of positions in the class structure do families currently occupy based on their migratory origin and ethnic self-identification? Table 2 provides a descriptive overview of this distribution for 2015–23, offering initial evidence of differentiated incorporation patterns across groups.

**Table 2:** Class position by migration background and ethnic descent (%). Individuals aged 25–65 residing in MABA, 2021 and 2023.

Class	Migration background and ethnic descent									
	Second generation born in MABA of European descent	Born in MABA to European migrant parents	Born in MABA to internal migrant parents and of European descent	Internal migrants of European descent	Second generation born in MABA with Mestizo descent	Born in MABA to internal migrant parents and of Mestizo descent	Internal migrants of Mestizo descent	Born in MABA to Latin American migrant parents	Latin American migrants	Total
I. Upper service class	12.8	18.64	14.65	12.28	5.79	6.49	1.35	6.49	5.81	10.53
II. Lower service class	22.6	21.19	20.38	12.28	16.53	12.99	6.76	17.24	6.98	17.85
IIIab. Routine non-manual class	22.8	17.8	23.25	15.79	19.42	19.48	5.41	20.69	13.95	20.07
IVab. Petty bourgeoisie	13.2	22.03	13.38	12.28	13.64	10.82	12.16	3.45	13.95	13.05
V + VI. Skilled working class	9.8	11.86	8.28	14.04	13.64	11.69	25.68	14.94	18.6	12
VIIab+IVc. Unskilled working class and farm workers	18.8	8.47	20.06	33.33	30.99	38.53	48.65	36.78	40.7	26.51
Total	100	100	100	100	100	100	100	100	100	100

Source: pooled surveys (21–23). Individuals aged 25–65 residing in MABA, 2021 and 2023.

Table 2 reveals^4^ marked differences in class position by family migratory origin and generation of migration to the Buenos Aires Metropolitan Area (MABA). Among families of European descent, both long-established residents and those with European migrant parents show a clear concentration in the upper-middle and middle classes (I-IV), indicating a sustained pattern of socioeconomic incorporation. Internal migrants of European descent occupy an intermediate position, maintaining partial continuity with the advantages historically associated with European descent.

In contrast, families of Mestizo and Latin American origin exhibit a stronger concentration in the working and lower strata (V–VII). For both groups, first-generation migrants have the highest proportions in the unskilled working class. Among their descendants, upward shifts to the skilled working class and the routine non-manual class can be observed; however, the overall class profiles remain substantially more disadvantaged than those of European-origin groups. These patterns are also reflected in Table A2, Supplementary Table 1, which presents the gross effects of the independent variables on the log(odds) of reaching each class relative to the unskilled working class.

**Figure 1:**
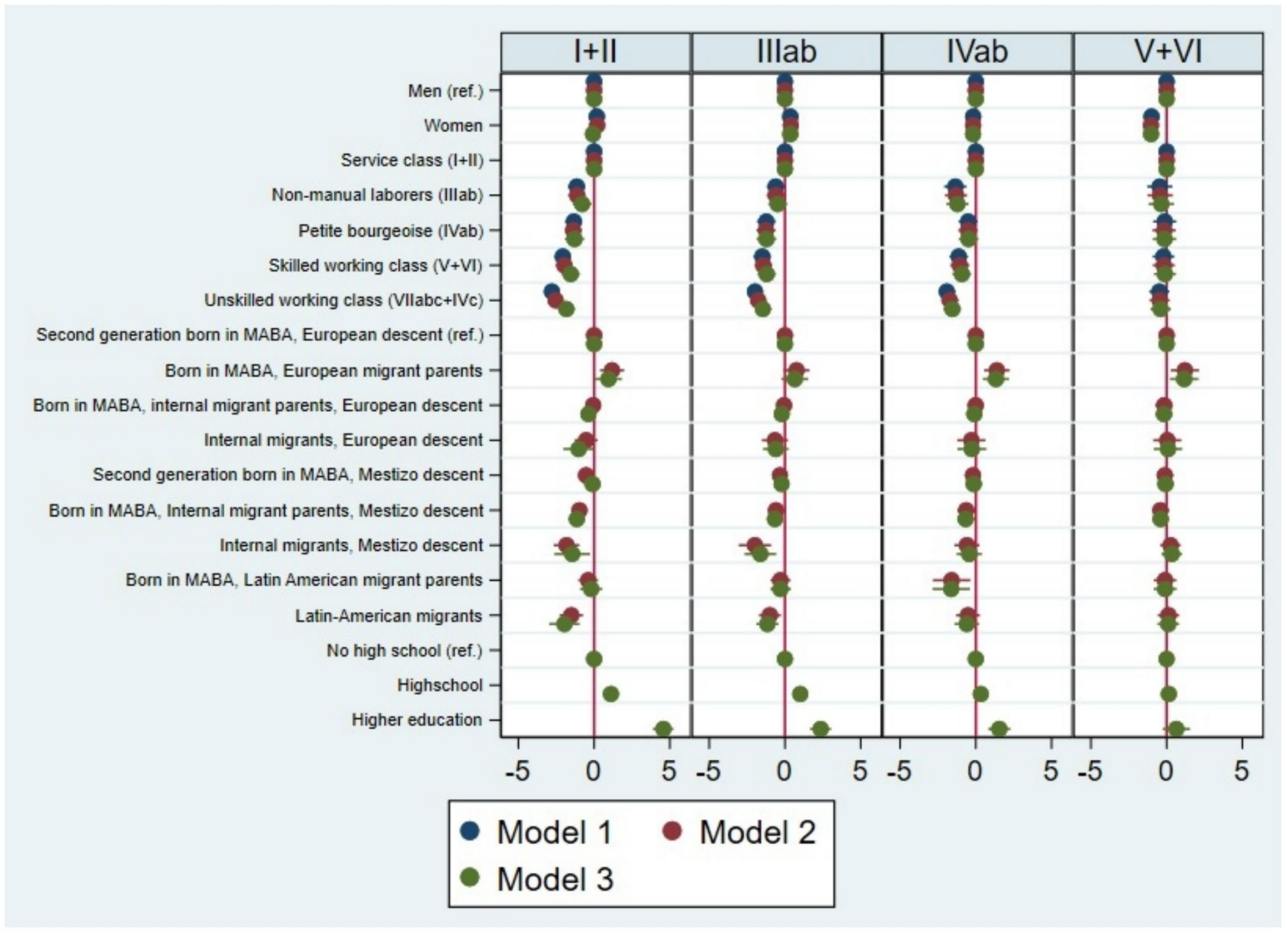
Coefficients for multinomial logistic regression models from Table A3, log(odds) of reaching each class destination (relative to Classes VIIab+IVc). Individuals aged 25–65 residing in MABA, 2021 and 2023. Source: pooled surveys (21–23).

Does the observed pattern of disadvantage in the class structure among internal migrants of mestizo descent, Latin American migrants, and their descendants persist once social class origin and educational level are taken into account? In other words, do ethnic origins outweigh class origins in shaping the life chances of the children of internal and Latin American migrants? To address this question, we estimate three multinomial logistic regression models (see Table A3) to assess the likelihood of reaching each class destination versus falling into or remaining in the unskilled working class (Figure 1). Table 3 shows goodness-of-fit statistics for the three estimated models.

**Table 3:** Goodness-of-fit statistics for multinomial regression models in Table A3 with class destinations as dependent variable (preferred model shown in boldface).

Model	L^2^	df	BIC
(1) Class origins + sex	320.74	24	5079.6
(2) 1 + Migration background and ethnic descent	426.43	56	5211.6
**(3) 2 + Education**	**921.44**	**64**	**4776.0**
Contrasts			
(2) (1)	−105.7	32	132
(3) (2)	−495.0	8	−435

Contrasts are significant at the 0,000 level. Source: pooled surveys (21–23).

Model 1 is a baseline model^4^, positing that class origins and sex significantly affect the log(odds) of attaining certain classes instead of class VII+IVc. Model 2 posits that the combined variable of migratory origin and ethnic descent influences the log(odds) of different paths of class mobility, net of the effects of class origin and sex. It tests the hypothesis that individuals from subaltern ethnic groups face fewer opportunities for upward mobility.

**Figure 2:**
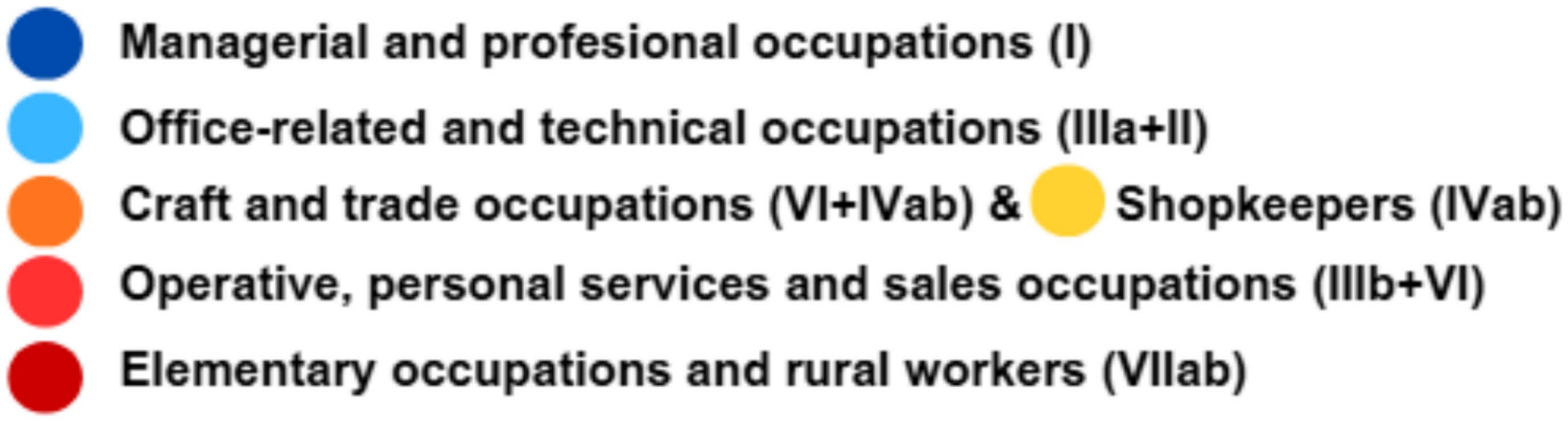
Levels for occupations in networks working class, VIIab, and IVc (source: Own elaboration).

Inspecting the LR test (105.7, *p* < 0.001) in Table 3, we observe that adding migratory origin and ethnic descent significantly improves model fit compared to Model 1 (also increasing pseudo-R^2^ from 0.062 to 0.081). The typology combining migratory origin and ethnic descent adds a significant effect on intergenerational class mobility pathways, net of class origin and sex (Figure 2).

When we compare the results of Model 2 with the gross effect of migratory origin and ethnic descent on the log(odds) of reaching each class relative to the unskilled working-class outcome (Table A2, Supplementary Table 1), we find that the inclusion of class origin attenuates the magnitude of inequalities, but the general pattern remains: subaltern groups continue to display lower chances for upward mobility into middle-class positions. This pattern suggests that some of the disadvantages faced by subaltern ethnic groups are associated with their later arrival in the MABA and their more frequent origins in lower-class backgrounds (i.e., the disadvantages of the offspring of Latin American migrants diminish). However, a net effect of migratory origin and ethnic descent persists for certain groups that cannot be explained by disparities in class origins (Model 2).

Model 3 adds educational attainment as a key factor influencing pathways of upward mobility, controlling for the ascriptive factors mentioned above. We introduce education under the hypothesis that some of the disadvantages faced by subaltern groups reflect cumulative inequalities associated with lower class origins and reduced educational attainment. As expected, schooling is strongly associated with class destinations. The likelihood-ratio test indicates that adding educational attainment significantly improves model fit compared to Model 2 (LR = 495.1, *p* < 0.001) (Table 3). In terms of overall goodness of fit, Model 3 outperforms the previous specifications, as indicated by the substantial increase in pseudo-R^2^ (from 0.081 to 0.176) and the lower BIC value (4776.07).

To summarize, we will consider the coefficients of Model 3, which provides a better overall fit than the previous models. The results show that controlling for class origin, sex, and educational attainment, the chances of attaining the service class and the routine non-manual class decrease for first-generation migrants of subaltern ethnic origins (internal migrants of Mestizo descent and Latin American migrants), as well as for the offspring of internal migrants of Mestizo descent (first-generation individuals born in MABA). In this regard, the chances of reaching the service class are also lower for the first generation of internal migrants of European descent. In contrast, when we add educational attainment, the disadvantages of second-generation individuals born in MABA of Mestizo descent disappear. On the other hand, the chances of reaching the service class, the petite bourgeoisie, and the skilled working class are higher for those born in MABA to parents of European migrants. In conclusion, these patterns indicate that part of the inequality in intergenerational social mobility opportunities faced by subaltern ethnic groups is linked to educational inequalities.

Gender also remains a relevant dimension of class attainment. Model 3 shows that women have lower chances than men of reaching the service class, the petite bourgeoisie, and the skilled working class (relative to remaining in the unskilled working class) but higher chances of attaining routine non-manual positions.

In sum, the regression models reveal that the combined variable of migratory background and ethnic descent is significantly associated with unequal opportunities to attain class destinations that represent different pathways of upward mobility compared to remaining in or falling to the unskilled working class. This association is partly related to the accumulation of disadvantages across previous generations, reflected in lower class origins and contemporary educational inequalities. Within this framework, the offspring of Latin American immigrants appear to experience some degree of partial equalization through educational attainment.

### A network analysis of class mobility at the occupational level

In this section, we deepen the analysis of the general trends studied with regression models by conducting a micro-level examination of intergenerational occupational pathways through social network analysis (SNA); while descriptive, this approach allows us to highlight occupation-specific types of reproduction for resources such as human capital (skills), cultural capital (tastes and aspirations), and social capital gained in occupation-specific networks (Jonsson et al., 2009, p. 7).

**Figure 3:**
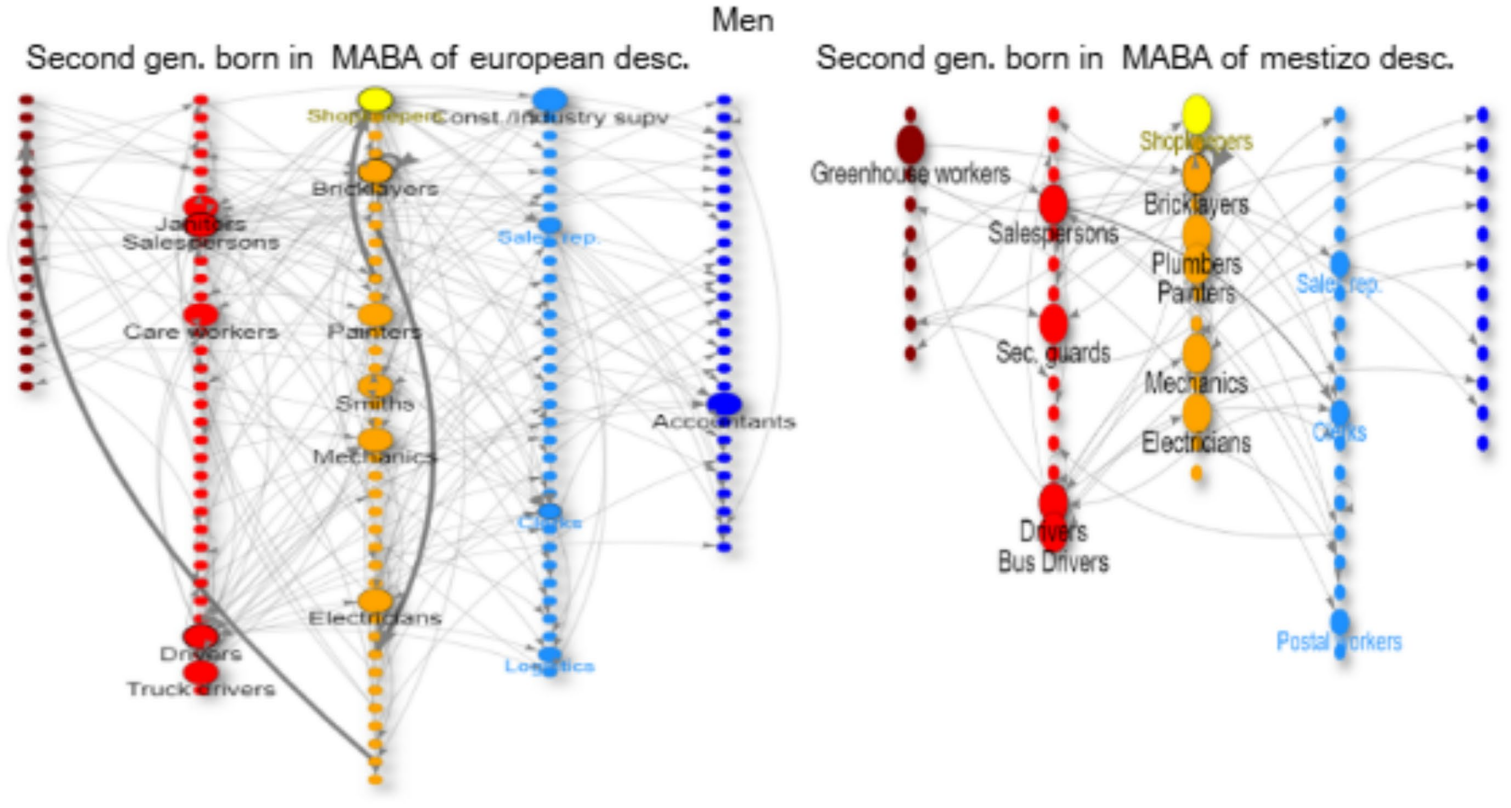
Intergenerational mobility network of key occupations among second-generation natives of the MABA by ethnic descent, men with working class origins. Source: pooled surveys (21–23).

**Figure 4:**
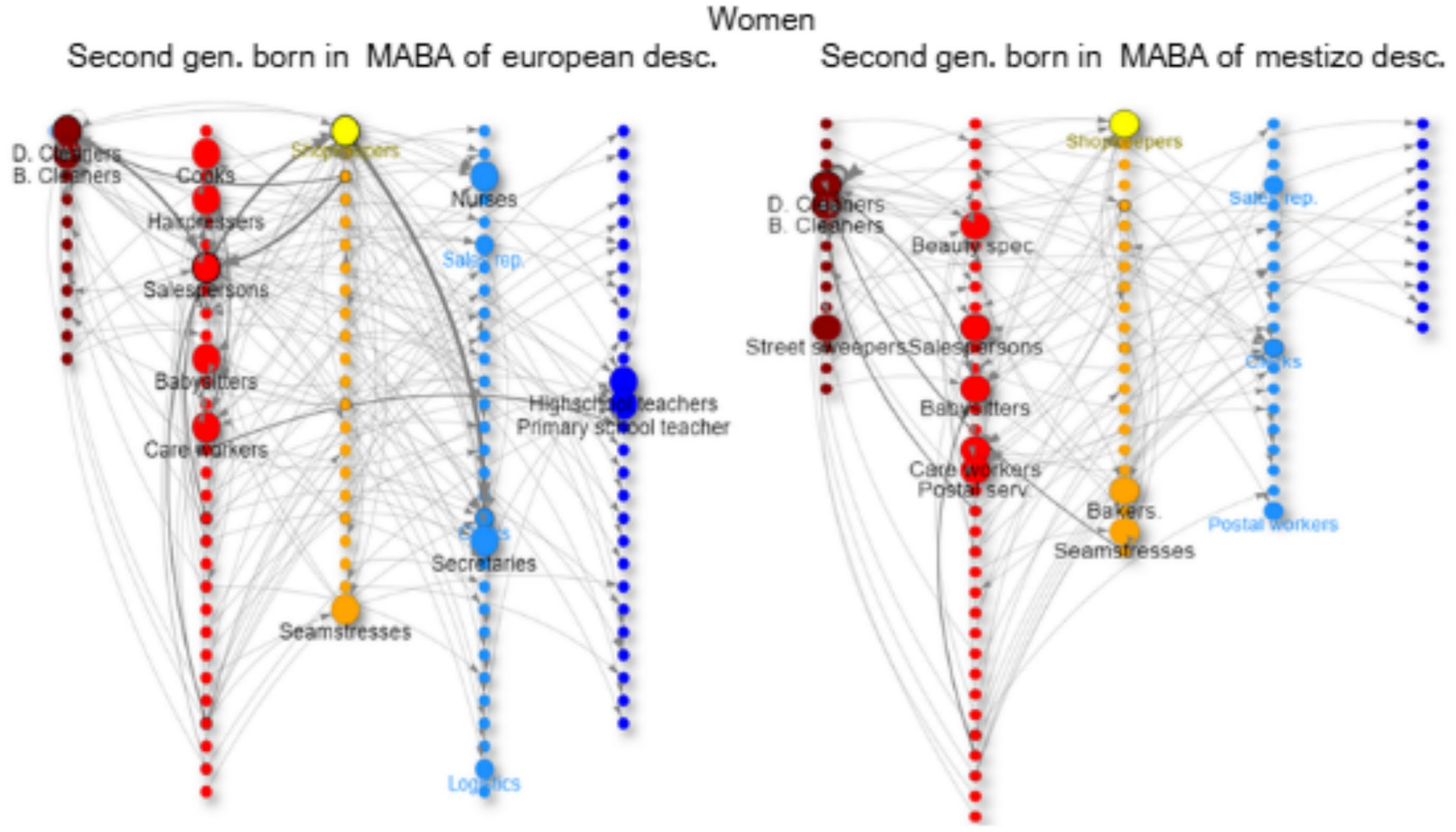
Intergenerational mobility network of key occupations among second-generation natives of the MABA by ethnic descent, women with working class. Source: pooled surveys (21–23).

**Figure 5:**
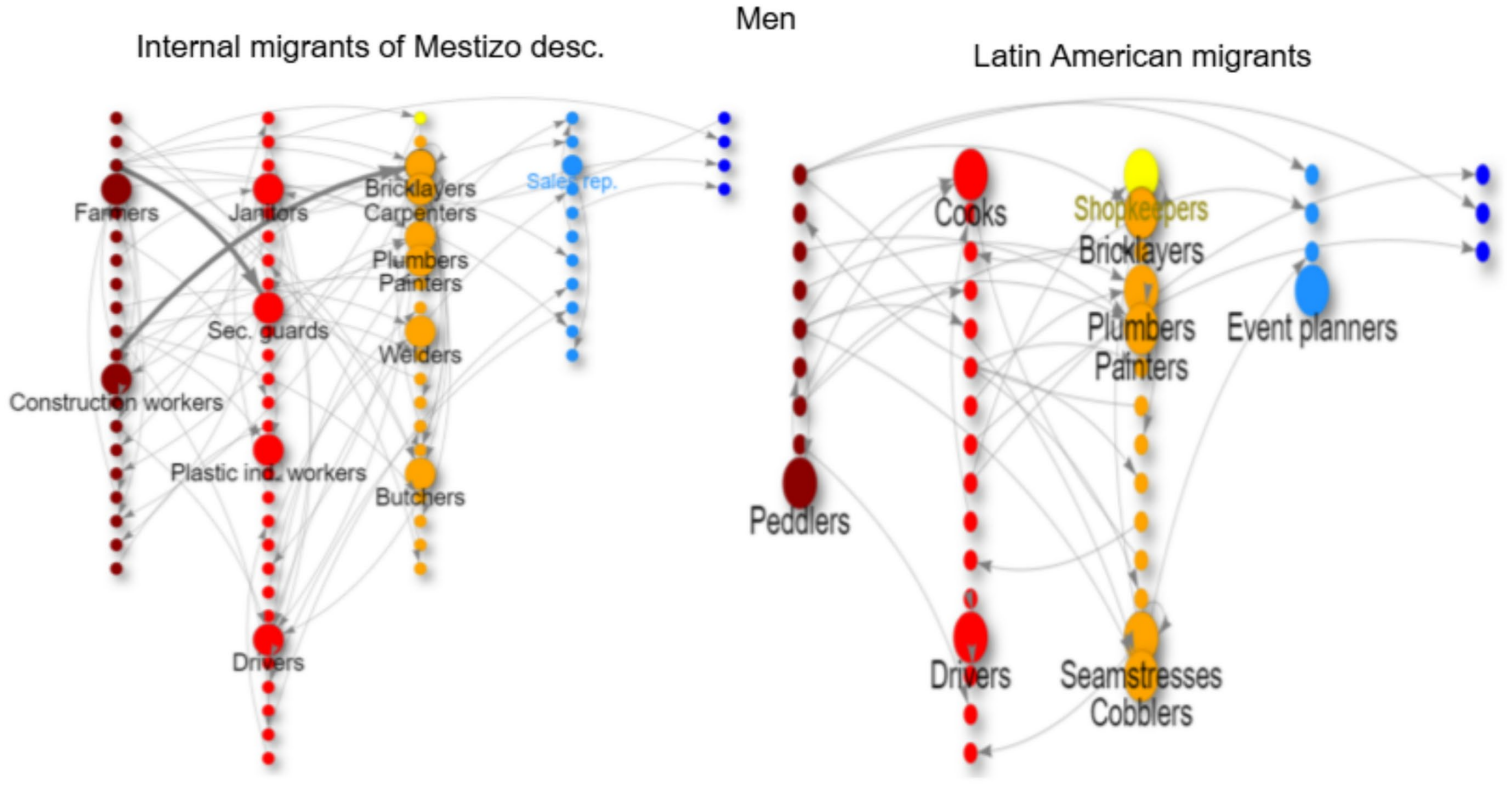
Intergenerational mobility network of key occupations among internal migrants of mestizo descent and Latin American migrants, men with working class origins. Source: pooled surveys (21–23).

**Figure 6:**
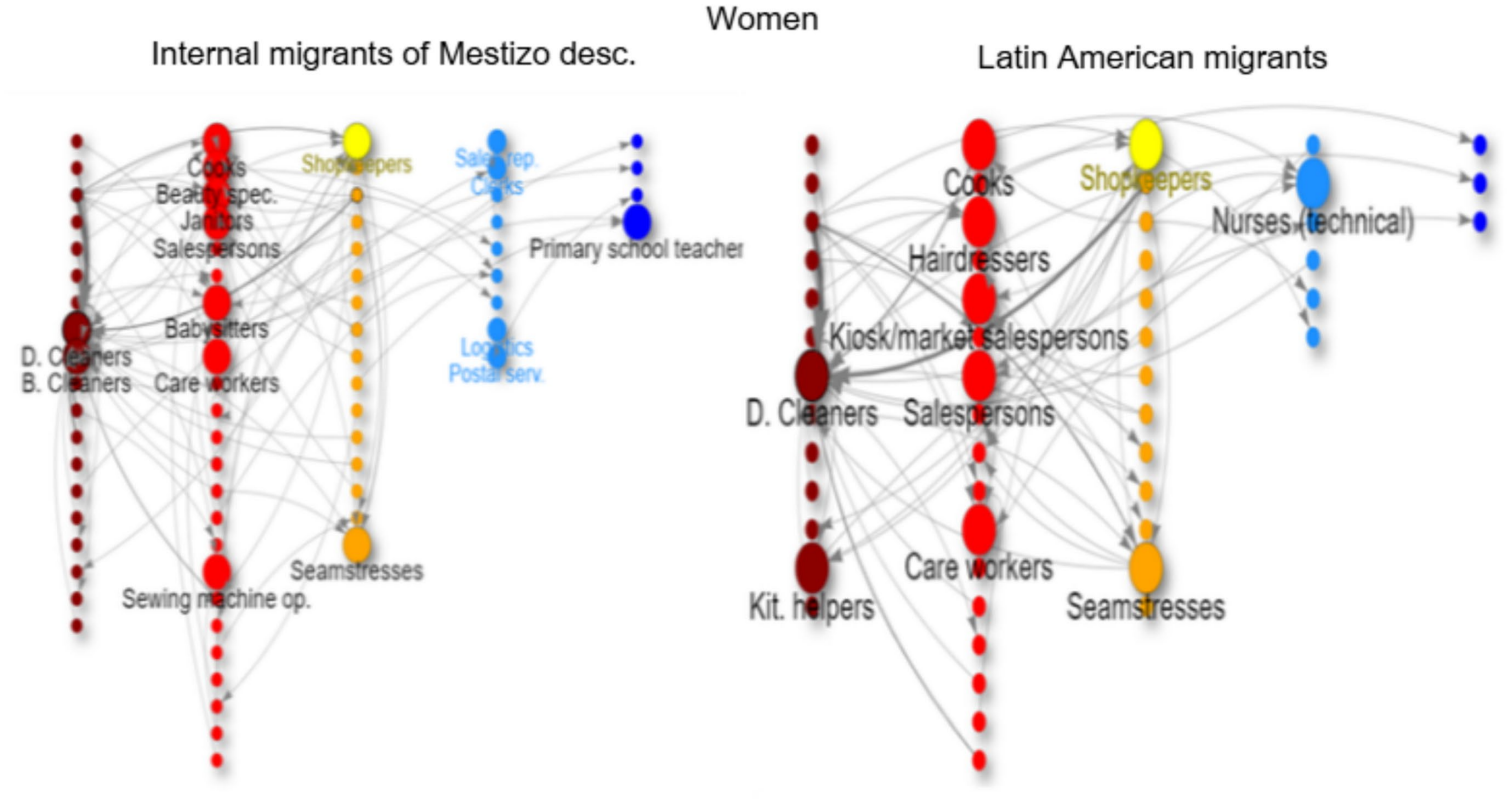
Intergenerational mobility network of key occupations among internal migrants of mestizo descent and Latin American migrants, women with working class origins. Source: pooled surveys (21–23).

**Figure 7:**
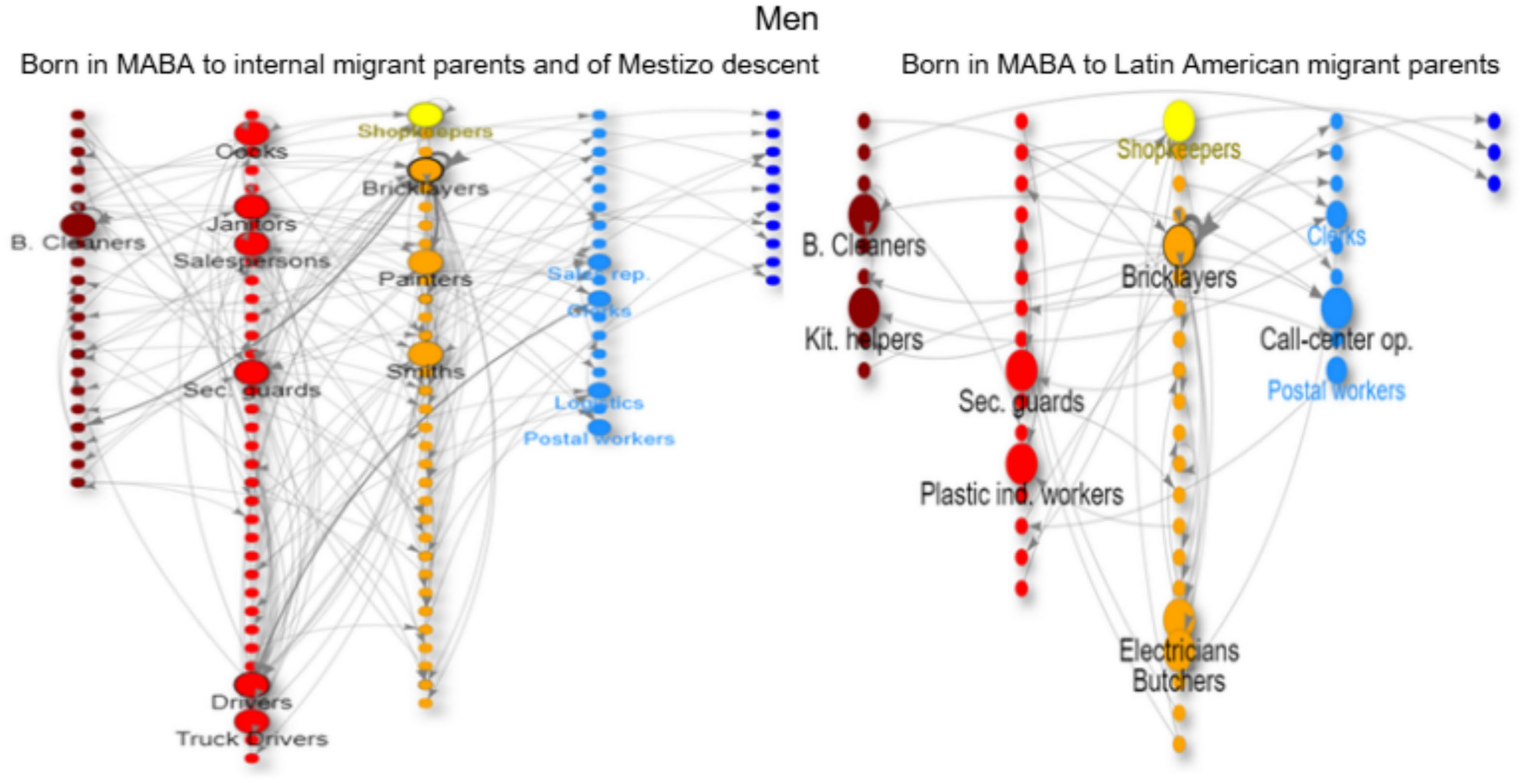
Intergenerational mobility network of key occupations among the offspring of internal migrants of mestizo descent and the offspring of Latin American migrants, men with working class origins (born in MABA). Source: pooled surveys (21–23).

**Figure 8:**
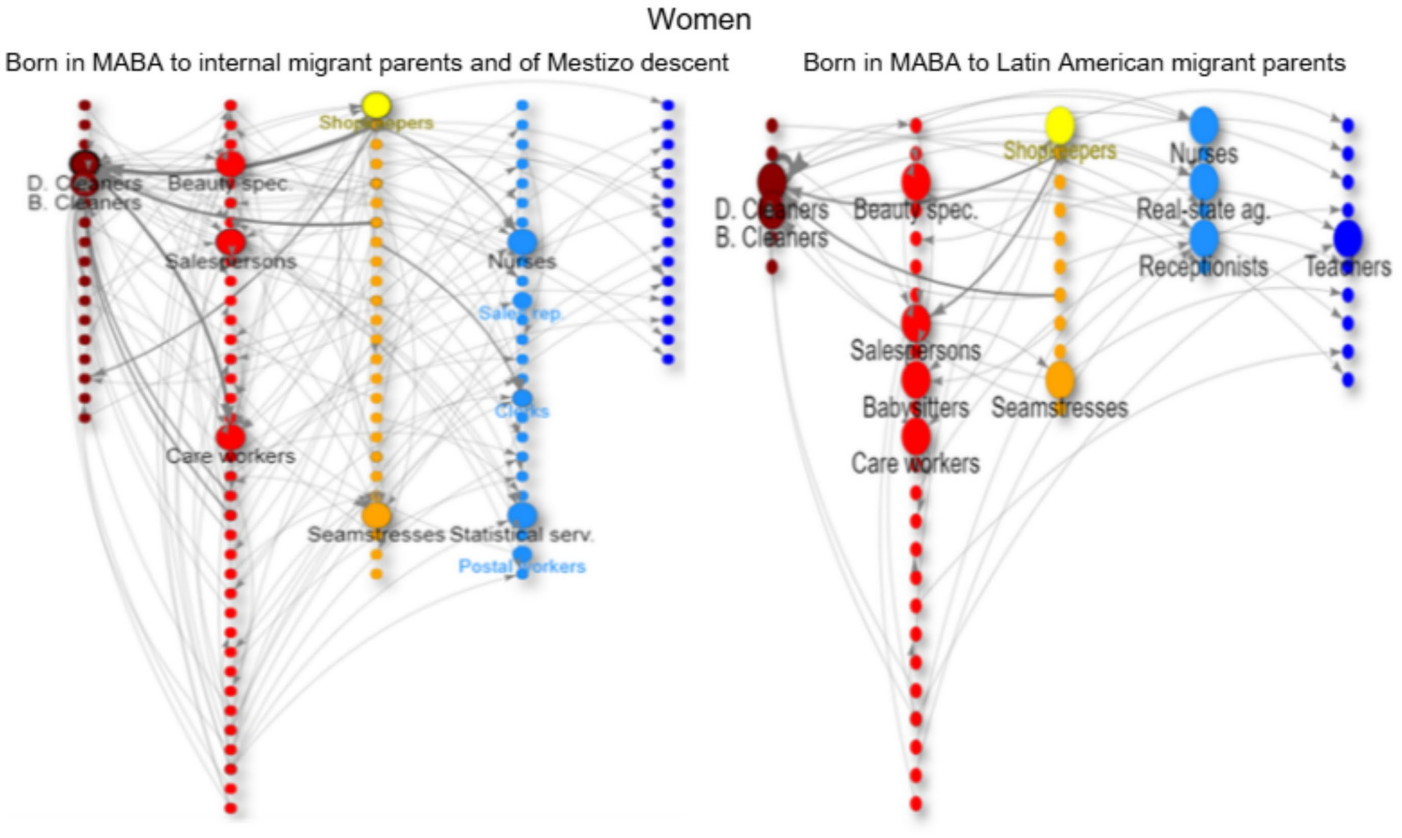
Intergenerational mobility network of key occupations among the offspring of internal migrants of mestizo descent and the offspring of Latin American migrants, women with working-class origins. (Born in MABA). Source: pooled surveys (21–23).

In the network graphs (Figures 3–8), nodes represent ISCO-08 occupations and are connected if there is an intergenerational transition from one to the other (from a father or mother to the respondent). Arrows indicate the direction of transitions (from father or mother to the respondent), and arrow width represents the weight of that transition in the survey. The *top nodes* (between 10 and 14 nodes with the highest in-degree centrality for each graph) are shown in a larger size and are labeled. Node colors represent an *approximation* of the social class position of each occupation following the EGP schema. Blue denotes managerial and professional occupations (service classes I); light blue represents office workers and technical occupations (IIIa+II); yellow corresponds to shopkeepers (ISCO 5221), used as a proxy for the petty bourgeoisie (IVab); orange indicates craft and trade workers (part of the skilled working class, although some may be self-employed or employers and would be part of the petty bourgeoisie); red represents operative-level workers, personal services, and sales occupations (a broad working-class category including IIIb and VI); and dark red denotes elementary occupations and rural workers (approximating the unskilled working class, VIIab and IVc). In other words, do ethnic origins trump class origins in shaping the life chances of the children of internal and Latin American migrants? To address this question, we estimate three multinomial logistic regression models^5^ (see Table A-3 in the Annex for model details) to assess the likelihood of reaching each class destination versus falling into or remaining in the unskilled working class (Figure 1). Table 3 shows goodness-of-fit statistics for the three estimated models.

To begin, we consider respondents who are the second generation in the MABA with European ancestry as a sort of *reference category* (as shown in the regression models). This is the largest category, totaling 27% of respondents and with the highest chances of upward class mobility.

The social origins of this group (for men and women) are typical of a *skilled* and *consolidated* working class. Many of the parents in this category are trade workers (mechanics, electricians, plumbers, smiths, builders, bricklayers, etc.), some are factory workers or drivers (cars, taxis, buses, trucks), two occupational groups that have high unionization rates and high salaries, and some are shopkeepers (mainly from family businesses, without employees). This group also includes many salespersons and some policemen, janitors, and cleaners.

The main *magnets* of class reproduction for men (red nodes) from this group are as drivers (mostly cars, with much lower incomes than unionized bus or truck drivers) and also trades such as bricklayers, mechanics, painters, electricians, smiths, etc (Figure 3). Instead, women work as salespersons and in other highly *feminized* occupations such as cleaners (domestic and otherwise), care workers (caregivers, babysitters), cooks, hairdressers, and seamstresses (Figure 4). Some of these trajectories represent downward mobility related to changes in the occupational structure in the last decades in Argentina due to a de-industrialization process.

This group has the highest levels of upward mobility (more blue and light blue nodes), and their main *springboard* is non-manual know-how (office clerks and shopkeepers).^6^ For men, there is a focus on occupations in logistics and as supervisors (Figure 3); this is tied to a shift from industry to service (transport and logistics) linked with the transition from an import-substitution industrialization model to a service-based economy. This is a pathway of intra-generational upward mobility (from shop-floor worker to supervisor/logistics handler). For women, there is a focus on teaching and health (nurses and other care work), as well as secretarial roles (Figure 4).

The second generation of residents in the MABA, but with mestizo ancestry, have lower opportunities for social mobility. They come from lower origins than their counterparts with European ancestry, with a higher number of parents who worked as bricklayers and domestic cleaners and fewer parents who worked as bus/truck drivers, factory workers, or in commerce as salespersons or shopkeepers. Similarly, their pathways for reproduction in the working class are of lower status, especially for women, where cleaners, babysitters, and caregivers are their main occupations (with lesser access to commerce) (Figure 4). For men, the main occupations are as bricklayers or drivers, including some trades (orange nodes: plumbers, mechanics, electricians, etc.) and some unskilled labor as gardeners or security guards (Figure 3). Both men and women have some upward mobility opportunities as shopkeepers (yellow) or office workers (light blue nodes: clerks, secretaries, etc.).

Internal migrants with mestizo ancestry from the working class come from low occupational origins (Figure 5). Many have rural backgrounds as farmers and animal breeders or work in unskilled agricultural, forestry, and other occupations such as cleaners and freight handlers. Men migrating to the metropolitan area often work in trades (orange nodes: bricklayers, plumbers, butchers, carpenters, painters) and in service occupations such as car drivers, security guards, or janitors (Figure 5). For women, the primary channel for employment is as cleaners (43% of them); some are involved in caregiving (babysitters and caregivers), while others have trades such as seamstresses, cooks, and beauty assistants (Figure 6). The chances of long-distance upward mobility for this group are almost nonexistent, with some short-distance mobility occurring through logistics and supervision for men and as shopkeepers or teachers for women.

Migrants from Latin American countries also have very low social origins, including a significant number of rural workers (farmers, breeders, unskilled agricultural workers, etc.), domestic cleaners, bricklayers, and textile workers (in factories, workshops, and as seamstresses). After arriving in the Metropolitan Area of Buenos Aires and entering the labor market, migrant men typically work in trades (bricklayers, cooks, painters, cobblers, plumbers, etc.) (Figure 5), while women primarily work as domestic cleaners (and also as seamstresses, salespersons, kitchen helpers, caregivers, etc.)^7^ (Figure 6). This group experiences some upward mobility (many moving from rural areas to urban centers or from unskilled labor to skilled trades), but very little long-distance upward mobility to the middle class; some women manage to become shopkeepers or enter health services as technical nurses (Figure 6).^8^

The offspring of internal migrants with mestizo ancestry also have low social origins (but not as low as their parents, who have already experienced rural–urban mobility); the prevalence of unskilled occupations such as janitors, cleaners, freight handlers, and security guards is much higher, along with trades (bricklayers, painters, etc.) associated with poorer working conditions (Figure 7). They have similar gendered occupations as channels of reproduction and mobility (logistics, clerks, and shopkeeping) as the second generation of natives in the MABA, but with lower chances of upward mobility (as indicated by the regression analysis).

The offspring of migrants from Latin American countries with working-class origins have similar occupations to their parents, focusing on trades and commerce for men (Figure 6) and cleaning and care work for women (Figure 8). Men tend to follow their *father’s footsteps,* achieving some short-range mobility, transitioning from construction workers to contractors (for example, Paraguayans) or accumulating capital through commerce (for example, Bolivians in horticulture). This group can even equalize their opportunities for long-range upward social mobility with the group of two generations in MABA when controlling for education. Men can work as office clerks or call-center workers (Figure 7), while women can also become teachers, nurses, shopkeepers, real estate agents, or even doctors (Figure 8). For women, there is a trend of *polarization* in the trajectories; they either experience downward mobility in precarious occupations such as domestic cleaning or care work, or they achieve upward mobility through tertiary education (nurses, medical technicians, etc.) (Figure 8).

## Conclusions and final reflections

In this paper, we analyzed intergenerational social mobility patterns from the working class by family migratory origin. Our research stands out from other work on this topic by using data from surveys that combine information on migratory origins with ethnic origins. This allowed us to differentiate between people who are the second generation born in the Metropolitan Area of Buenos Aires, the sons and daughters of migrants, and migrants themselves (both internal and Latin American). Additionally, for Argentinians, we differentiated between those of European descent and subaltern groups, specifically those of mestizo descent.

Our results reveal clear inequalities in class attainment by family migratory origin. Some groups face disadvantages even when controlling for class origins and education (internal migrants and their offspring of mestizo descent and Latin American migrants). These subaltern groups have fewer opportunities for upward social mobility, both in terms of long-range pathways into the service class (more markedly) and short-range movements into routine non-manual occupations and the petty bourgeoisie (less markedly). Meanwhile, the second generation of MABA residents of mestizo descent and the offspring of Latin American migrants are able to *reverse* these *disadvantages* and equalize their opportunities for upward mobility with the more privileged group.

These outcomes suggest that ethno-racial inequality is associated with differences in mobility chances, but it should not be considered the only factor at play. Rather, it can be understood in relation to the interplay between shifts in the structure of opportunities, the process of racialization, the intergenerational transmission of aspirations for higher educational and occupational attainment, and specific forms of parental support more frequently observed among external migrant families. In line with this, Zuccotti (2023) finds that young people of Latin American migrant origin (from Paraguay, Bolivia, and Peru) with low parental educational backgrounds have achieved better educational outcomes than native Argentinians with similar origins.

Based on this, we analyzed specific occupational *pathways* for intergenerational mobility or reproduction using social network analysis. The main patterns reveal high levels of class reproduction, with only limited upward mobility flows from the upper (or consolidated) segments of the working class into the middle class. Such mobility is mainly directed toward routine non-manual occupations, with restricted access to the service class in professional positions. A few specific gendered *springboards* for upward mobility can be observed, particularly in health and education for women and in logistics for men.^9^

Generally, we observe a common pattern of low opportunities for long-distance upward social mobility from the working class to the upper middle class, with subtle differences between groups in the *pathways* followed. We see differences in the *degree* of mobility achieved (subaltern groups have less upward mobility); respondents of European origin and those with two or more generations in the MABA come from higher and more consolidated working-class origins (fathers with skilled, stable, and unionized occupations), making it easier for their offspring to reproduce this status. The second generation of natives in the MABA with European descent follow more traditional mobility paths. Although they were affected by deindustrialization, they tend to achieve class reproduction in skilled trades and service occupations, as well as upward mobility into supervisory positions, logistics-related occupations, office work, and some liberal professions. The two generations of natives in the MABA of *mestizo* descent started from lower social origins and were more strongly affected by deindustrialization. Their trajectories are characterized by higher levels of self-employment and employment in low-tier service occupations, with more limited long-range upward mobility. Finally, natives in the MABA whose parents were internal migrants of mestizo descent followed a similar path to the latter group but with even fewer opportunities for upward mobility.

On the other hand, the offspring of Latin American migrants follow family paths in ethnic economic enclaves (construction and commerce) with class reproduction but some economic upward mobility through the accumulation of capital. For women, education is key to achieving short-range upward mobility. The offspring of internal migrants of mestizo descent have a stronger focus on education (teaching), while the daughters of Latin American migrants focus more on health (nursing, technicians, etc.). Nevertheless, the daughters of Latin American migrants experience *polarized trajectories*; those who do not achieve higher education face downward mobility through precarious occupations (cleaning, care work, and textile work in informal workshops), while the daughters of internal migrants of mestizo descent have a wider range of opportunities, ranging from lower occupations (cleaning and care work) to factory work (mainly textile and food industry), service occupations (commerce, beauty, and hairdressing), office work, and teaching through higher education.

Results show evidence supporting the importance of the “opportunity structure” (Dalle, 2025). The first migrant flows of European origin arrived at the MABA when the class structure was still in the process of formation, and the last arrived during the industrialization process. These families of European origin also relied on the dense social networks of their communities (Italian, Spanish, Jewish), which contributed to their collective trajectories of upward mobility by providing support, housing, employment, etc. From a stable position in the working class or within the consolidated segment of the working class, these families were able to transmit a wider set of upward social mobility opportunities to their descendants through trades and skilled manual labor in the formal and unionized segment of the labor market.

The first flows of internal migrants (partly of mestizo descent) also began during the industrialization period. Their descendants (in our work: those with two generations in the MABA and of mestizo descent) had fewer opportunities for social mobility. This group settled heavily in the Conurban Area of Buenos Aires and experienced upward mobility in a context marked by the expansion of industrialization and economic growth. While they had fewer chances than those of European descent, they were able to reach similar levels of mobility when controlling for education.

On the other hand, the most recent flows of internal migrants of mestizo descent and migrants from Latin American countries arrived in the region at a time when society had become more segmented and less dynamic. Shortly after their arrival in the metropolis, they faced a context characterized by deindustrialization and recurring economic crises, which severely constrained their opportunities for upward mobility or for maintaining positions within the skilled and more integrated segments of the working class. Consequently, these groups had a relatively high presence in unskilled service activities (domestic cleaning and care work for women) and construction (for men), mostly within the informal labor sector linked to poverty conditions. A considerable proportion settled in informal settlements (both in the City of Buenos Aires and in the Conurban Area), where precarious employment and structural unemployment are prevalent, and sociability unfolds under conditions of scarcity, associated with short-term orientations toward fulfilling basic needs.

The sons and daughters of Latin American migrants achieve equal opportunities for upward mobility, when controlling for education, with gendered patterns. For men, this is achieved through commerce in certain occupational niches, with Bolivians dedicated to horticultural activities and small textile workshops, and Paraguayans engaged in construction and popular retail commerce. Women achieve mobility through higher education, mainly in occupations in health, care work, and teaching. This pattern is associated with a combination of ethnic capital, the formation of dense networks and economic enclaves where communities have gained vertical control of the production and retail process, and the motivations for improvement held by migrants along with the expectations of upward mobility they set for their offspring.

What are the policy implications of the patterns observed? How can the intergenerational reproduction of class inequality among subaltern migrant-origin groups be effectively disrupted? A closer examination of our findings indicates that a substantial part of the inequality in opportunities for upward social mobility among migrant groups of subaltern ethnic origin and their descendants is associated with educational attainment, which in turn reflects unequal educational opportunities. These groups tend to originate from lower social backgrounds, achieve lower levels of education, and attain class positions of lower status. When controlling for educational level, inequalities in upward mobility decrease, although certain groups continue to face disadvantages.

This has important implications for public policy. Effectively addressing inequalities linked to ethno-racial origins requires action on at least three fronts. First, the development of affirmative policies aimed at reversing both historical and contemporary inequalities associated with discrimination is crucial (Solís and Güémez, 2021), as illustrated by experiences such as Brazil. In this regard, expanding scholarship and support programs in secondary schools and universities for students from subaltern ethnic groups is key. Access to higher levels of education mitigates a substantial part of inherited disadvantages that tend to accumulate over the life course. Next, the implementation of effective anti-discrimination policies is necessary to address persistent discriminatory practices that hinder access to employment opportunities.

Second, a crucial component in fostering upward mobility among subaltern ethnic groups, which are largely characterized by working-class origins, is linked to sustained economic development and policies oriented toward greater equality of conditions between classes. This involves development policies aimed at upgrading the class structure, including the expansion of skilled manual jobs and stable formal employment, progressive income redistribution, investment in infrastructure in disadvantaged neighborhoods, and the continuous improvement of the quality of public education and health services (Hout and Di Prete, 2006; Beller and Hout, 2006; Goldthorpe, 2016; ILO, 2008).

Finally, and centrally, policies aimed at reducing the labor market segmentation of women in domestic and care work are essential. This includes promoting women’s employment in skilled sectors, expanding public and formal private care provision, and formalizing employment in private households (Esping Andersen, 2014). Such measures are likely to be associated with higher levels of upward mobility from the working class and with lower levels of intersectional inequalities that disproportionately affect working-class women from racialized ethnic groups.

## Data Availability

The data analyzed in this study is subject to the following licenses/restrictions: requests to access these datasets should be directed to pablodalle80@hotmail.com.

## Footnotes

1 Currently, in Argentina, “criollo” refers to the population that has several generations of Argentine ancestry. It is typically associated with the mestizo population, characterized by mixed European (usually Spaniard) and Indigenous descent but without recent European migratory roots (Dalle, 2025). This usage implies notions of mestizaje (in contrast to “gringo” settlers) and also operates as a social reference to belonging to the popular sectors (Grimson and Karasik, 2021, p. 22).

2 To assess the comparability of intergenerational mobility patterns across surveys, we compute Cramér’s V for the cross-tables of class origins and class destinations for each survey. The resulting values (2021 = 0.18, and 2023 = 0.17) indicate highly similar associations across data sources, suggesting that the underlying mobility patterns are stable over a short time span.

3 Regarding the influence of COVID-19, in the 2021 survey, information on class position based on occupational indicators was collected for two points in time: the first months of 2020 (prior to the COVID-19 lockdown) and the time of the survey (November–December 2021). This allows us to observe occupational positions both before and after the pandemic period. Evidence from a previous study indicates that there were no major shifts in the overall class structure during this period, as most workers returned to similar occupational positions after the pandemic (Benza et al., 2022). In our study, class destination is measured using respondents’ current class position at the time of the survey (end of 2021).

4 Data and code for replication is available at: https://github.com/JoaquinCarrascosa/Dalle-Carrascosa-2026.

5 We also conducted a sensitivity analysis, examining the effects of migratory origin and ethnic background separately, as well as their interaction. The results indicate that both variables have significant effects, whereas the interaction term is not statistically significant (based on the likelihood ratio test) (Table A-4, supplementary Table 2). We opt to present the results using the combined variable, because it shows a clearer pattern, particularly the disadvantages faced by internal migrants of Mestizo descent and their descendants.

6 We consider many cases of shopkeepers as upward mobility because they mostly come from occupations of lower status and in some cases are also employers (IVa) while shopkeepers’ parents are all self-employed (IVb).

7 Paraguayan migrants are a typical case of this type of migration, where women work mainly as domestic cleaners or care occupations such as babysitters or carepersons while men work in construction (bricklayers, stonemasons, painters, etc.).

8 For Bolivian migrants and their offspring, there has been a process of upward economic mobility for horticulturists denominated “the Bolivian ladder” where migrants (with their families and larger communities) scale positions in the social structure from working the land in the peripheral zones of the Metropolitan Area of Buenos Aires to accessing the land and controlling supply chains and commercialization (Benencia, 2012).

9 In this paper we analyze mobility in individual terms (in many cases from father to daughter); data analyzing couple formation shows a trend of class homophily where women with these occupations have partners with masculinized working-class occupations mentioned here (bricklayers, plumbers, security guards, etc.) (Carrascosa, 2023).

## Appendix

**Table A3:** Multinomial logistic regression models for the log (odds) of reaching each class destination (relative to Classes VIIab+IVc). Individuals aged 25–65 residing in MABA, 2021 and 2023.

	**(ref. VIIab+IVc)**											
**Variables**	**I+II**	**IIIab**	**IVc**	**V+VI**	**I+II**	**IIIab**	IVc	**V+VI**	**I+II**	**IIIab**	**IVabc**	**V+VI**
**Sex**												
Men (ref)	–	–	–	–	–	–	–	–	–	–	–	–
Women	0.18	0.35*	–0.17	–1.00***	0.20	0.38*	–0.18	–1.03***	–0.08	0.35*	–0.18	–1.03***
	(0.14)	(0.15)	(0.17)	(0.18)	(0.15)	(0.16)	(0.17)	(0.18)	(0.17)	(0.16)	(0.17)	(0.18)
**Class origins**												
Service class (I+II) (ref.)	–	–	–	–	–	–	–	–	–	–	–	–
Non-manual laborers (IIIab)	–1.14***	–0.61*	–1.36***	–0.46	–1.12***	–0.61*	–1.30***	–0.43	–0.79*	–0.47	–1.21**	–0.36
	(0.29)	(0.30)	(0.37)	(0.42)	(0.29)	(0.31)	(0.38)	(0.43)	(0.32)	(0.31)	(0.38)	(0.43)
Petite bourgeoisie (IVab)	–1.34***	–1.23***	–0.51	–0.13	–1.38***	–1.25***	–0.52	–0.16	–1.29***	–1.22***	–0.48	–0.14
	(0.28)	(0.31)	(0.32)	(0.39)	(0.29)	(0.32)	(0.32)	(0.39)	(0.32)	(0.32)	(0.33)	(0.40)
Skilled working class (V+VI)	–2.07***	–1.50***	–1.13***	–0.21	–1.96***	–1.43***	–1.06***	–0.19	–1.55***	–1.21***	–0.92**	–0.11
	(0.27)	(0.29)	(0.31)	(0.37)	(0.28)	(0.30)	(0.32)	(0.37)	(0.31)	(0.30)	(0.32)	(0.38)
Unskilled working class and farm workers (VIIab+IVc)	–2.79***	–1.98***	–1.91***	–0.48	–2.54***	–1.77***	–1.72***	–0.46	–1.83***	–1.46***	–1.55***	–0.40
	(0.25)	(0.26)	(0.29)	(0.34)	(0.25)	(0.27)	(0.30)	(0.34)	(0.28)	(0.28)	(0.30)	(0.35)
**Migration background and ethnic descent**												
Second generation born in MABA, European descent (ref.)												
Born in MABA, European migrant parents					1.18**	0.76	1.39**	1.20*	0.95*	0.65	1.33**	1.16*
					(0.40)	(0.43)	(0.43)	(0.47)	(0.43)	(0.44)	(0.43)	(0.47)
Born in MABA, internal migrant parents, European descent					–0.06	–0.06	–0.01	–0.16	–0.37	–0.21	–0.10	–0.17
					(0.21)	(0.23)	(0.26)	(0.30)	(0.25)	(0.24)	(0.26)	(0.30)
Internal migrants, European descent					–0.52	–0.66	–0.29	0.04	–1.02*	–0.60	–0.26	0.10
					(0.40)	(0.44)	(0.48)	(0.47)	(0.51)	(0.46)	(0.49)	(0.47)
Second generation born in MABA, Mestizo descent					–0.53*	–0.33	–0.19	–0.12	–0.10	–0.22	–0.14	–0.07
					(0.23)	(0.24)	(0.27)	(0.28)	(0.26)	(0.25)	(0.27)	(0.28)
Born in MABA, Internal migrant parents, Mestizo descent					–0.97***	–0.59*	–0.64*	–0.41	–1.14***	–0.66**	–0.67*	–0.40
					(0.24)	(0.24)	(0.29)	(0.29)	(0.28)	(0.25)	(0.29)	(0.29)
Internal migrants, Mestizo descent					–1.82***	–1.98***	–0.58	0.25	–1.45*	–1.63**	–0.43	0.34
					(0.48)	(0.55)	(0.42)	(0.35)	(0.59)	(0.56)	(0.42)	(0.35)
Born in MABA, Latin American migrant parents					–0.39	–0.30	–1.60*	–0.10	–0.20	–0.29	–1.62*	–0.10
					(0.32)	(0.34)	(0.63)	(0.39)	(0.37)	(0.34)	(0.63)	(0.38)
Latin-American migrants					–1.50***	–1.00**	–0.52	0.12	–1.96***	–1.16**	–0.60	0.11
**Education**												
No high school (ref)												
Highschool									1.11***	1.01***	0.33	0.14
									(0.20)	(0.17)	(0.19)	(0.18)
Higher education									4.57***	2.36***	1.55***	0.64
									(0.35)	(0.35)	(0.38)	(0.47)
**Constant**	1.68***	0.81***	0.55*	0.08	1.96***	1.05***	0.72*	0.04	0.21	0.18	0.38	–0.11
	(0.22)	(0.24)	(0.26)	(0.30)	(0.25)	(0.27)	(0.29)	(0.35)	(0.31)	(0.30)	(0.32)	(0.37)
**Pseudo R2**	0,061				0,081				0,176			
Observations	1638	1638	1638	1638	1638	1638	1638	1638	1638	1638	1638	1638
Lrtests	Lrtest M1 vs M2: LR chi2(32) = 105,7 | Prob > chi2 = 0,000 Lrtest M2 vs M3: LR chi2(8) = 495,1 | Prob > chi2 = 0,000											

Robust standard errors in parentheses. ****p* <0.001, ***p* <0.01, **p* <0.05. Source: pooled surveys (21–23).
