# Supplementary material for: Can cumulative disadvantages be reversed? Class attainment and a network analysis of intergenerational occupational pathways by migratory origin in Buenos Aires, Argentina
Source: Front Sociol. 2026 May 21;11:1755911. doi: 10.3389/fsoc.2026.1755911 (PMC13233345; doi:10.3389/fsoc.2026.1755911)
Supplement: Supplementary file 1 [file Table_1.docx]

**Appendix**

Table A-1. Migratory origin and ethnicity (%, total sample).

Individuals aged 18+ residing in MABA, 2021–2023.

|  | **Ethnicity** | | |
| --- | --- | --- | --- |
| **Migratory Origin** | Mestizo/indigenous | European (+arab and Jewish) | Total |
| Born in MABA from parents born in MABA | 15.5 | 29.1 | 44.7 |
| Born in MABA from overseas migrant parents | 1.0* | 6.2 | 6.2 |
| Born in MABA from internal migrant parents | 13.6 | 17.6 | 31.1 |
| Internal migrants | 4.1 | 3.0 | 7.11 |
| Born in MABA from Latin American migrant parents | 3.8 | 1.9* | 5.7 |
| Latin American migrants | 3.5 | 1.6* | 5.1 |
|  | 41.5 | 58.5 | 100 |
| Total | | | 1926 |

* Groups representing less than 2% of the total sample were collapsed into the predominant ethnic background category of each migratory group to ensure sufficient cell sizes for multivariate analysis.

Source: pooled surveys (21-23).

Table A-2. Gross effects for multinomial regression models on class destinations for each independent variable. Individuals aged 25–65 residing in MABA, 2021 and 2023

|  | **Model 1 (ref. VIIab)** | | | |
| --- | --- | --- | --- | --- |
| **Variables** | **I+II** | **IIIab** | **IVabc** | **V+VI** |
| **Class origins** |  |  |  |  |
| Service class (I+II) (ref.) | - | - | - | - |
| Non-manual laborers (IIIab) | -1.06*** | -0.52 | -1.25*** | -0.57 |
|  | (0.28) | (0.30) | (0.36) | (0.41) |
| Petite bourgeoisie (IVab) | -1.41*** | -1.24*** | -0.58 | -0.23 |
|  | (0.27) | (0.30) | (0.31) | (0.37) |
| Skilled working class (V+VI) | -2.05*** | -1.42*** | -1.09*** | -0.39 |
|  | (0.27) | (0.28) | (0.31) | (0.35) |
| Unskilled working class (VIIab) | -2.72*** | -1.85*** | -1.86*** | -0.61 |
|  | (0.24) | (0.25) | (0.28) | (0.32) |
| **Constant** | 1.78*** | 0.99*** | 0.49* | -0.30 |
|  | (0.21) | (0.23) | (0.24) | (0.30) |
| **Sex** |  |  |  |  |
| Men (ref.) | - | - | - | - |
| Women | 0.05 | 0.25 | -0.24 | -1.01*** |
|  | (0.13) | (0.14) | (0.16) | (0.17) |
| **Constant** | -0.00 | -0.45*** | -0.61*** | -0.35** |
|  | (0.10) | (0.11) | (0.12) | (0.11) |
| **Migration background and ethnic descent** |  |  |  |  |
| Second generation born in MABA, European descent (ref.) | - | - | - | - |
| Born in MABA, European migrant parents | 0.91* | 0.55 | 1.31** | 0.99* |
|  | (0.37) | (0.41) | (0.41) | (0.45) |
| Born in MABA, internal migrant parents, European descent | -0.08 | -0.05 | -0.05 | -0.23 |
|  | (0.20) | (0.22) | (0.26) | (0.29) |
| Internal migrants, European descent | -0.94* | -0.94* | -0.64 | -0.21 |
|  | (0.37) | (0.43) | (0.47) | (0.46) |
| Second generation born in MABA, Mestizo descent | -0.96*** | -0.66** | -0.47 | -0.17 |
|  | (0.22) | (0.23) | (0.26) | (0.27) |
| Born in MABA, Internal migrant parents, Mestizo descent | -1.31*** | -0.87*** | -0.92*** | -0.54 |
|  | (0.22) | (0.23) | (0.28) | (0.28) |
| Internal migrants, Mestizo descent | -2.42*** | -2.39*** | -1.03* | 0.01 |
|  | (0.46) | (0.55) | (0.41) | (0.33) |
| Born in MABA, **Latin American migrant parents** | -1.05*** | -0.77* | -2.01** | -0.25 |
|  | (0.31) | (0.33) | (0.62) | (0.37) |
| Latin-American migrants | -1.79*** | -1.26*** | -0.72 | -0.13 |
|  | (0.37) | (0.36) | (0.37) | (0.35) |
| **Constant** | 0.63*** | 0.19 | -0.35* | -0.65*** |
|  | (0.13) | (0.14) | (0.16) | (0.18) |
| **Education** |  |  |  |  |
| No high school (ref) | - | - | -- | - |
| Highschool | 1.34*** | 1.16*** | 0.56*** | 0.21 |
|  | (0.19) | (0.16) | (0.17) | (0.17) |
| Higher education | 4.93*** | 2.68*** | 2.00*** | 0.61 |
|  | (0.33) | (0.34) | (0.36) | (0.45) |
| **Constant** | -1.68*** | -1.09*** | -1.11*** | -0.90*** |
|  | (0.15) | (0.12) | (0.12) | (0.11) |
| **Pseudo R2 Class origins** | 0,04 | | | |
| **Pseudo R2 Sex** | 0,01 | | | |
| **Pseudo R2 Migration background and ethnic descent** | 0,033 | | | |
| **Pseudo R2 Education** | 0,124 | | | |
| Observations | 1638 | 1638 | 1638 | 1638 |

Source: pooled surveys (21-23).
